# Mitochondria as oncotarget: a comparison between the tetracycline analogs doxycycline and COL-3

**DOI:** 10.18632/oncotarget.26107

**Published:** 2018-09-18

**Authors:** Margherita Protasoni, Albert M. Kroon, Jan-Willem Taanman

**Affiliations:** ^1^ Department of Clinical and Movement Neurosciences, Institute of Neurology, University College London, London, NW3 2PF, UK

**Keywords:** cancer, COL-3, doxycycline, mitochondria, tetracycline

## Abstract

Tetracyclines have anticancer properties in addition to their well-known antibacterial properties. It has been proposed that tetracyclines slow metastasis and angiogenesis through inhibition of matrix metalloproteinases. However, we believe that the anticancer effect of tetracyclines is due to their inhibition of mitochondrial protein synthesis, resulting in a decrease of the mitochondrial energy generating capacity. Several groups have developed analogs that are void of antibacterial action. An example is COL-3, which is currently tested for its anticancer effects in clinical trials. We have undertaken a comparative study of the tetracycline analogs COL-3 and doxycycline, which has an antibacterial function, to further investigate the role of the mitochondrial energy generating capacity in the anticancer mechanism and, thereby, evaluate the usefulness of mitochondria as an oncotarget. Our experiments with cultures of the human A549, COLO357 and HT29 cancer cells and fibroblasts indicated that COL-3 is significantly more cytotoxic than doxycycline. Mitochondrial translation assays demonstrated that COL-3 has retained its inhibitory effect on mitochondrial protein synthesis. Both drugs caused a severe decrease in the levels of mitochondrially encoded cytochrome-c oxidase subunits and cytochrome-c oxidase activity. In addition, COL-3 produced a marked drop in the level of nuclear-encoded succinate dehydrogenase subunit A and citrate synthase activity, indicating that COL-3 has multiple inhibitory effects. Contrary to COL-3, the anticancer action of doxycycline appears to be based specifically on inhibition of mitochondrial protein synthesis, which is thought to affect rapidly proliferating cancer cells more than healthy tissue. Doxycycline is likely to cause less side effects that COL-3.

## INTRODUCTION

**Figure 1:**
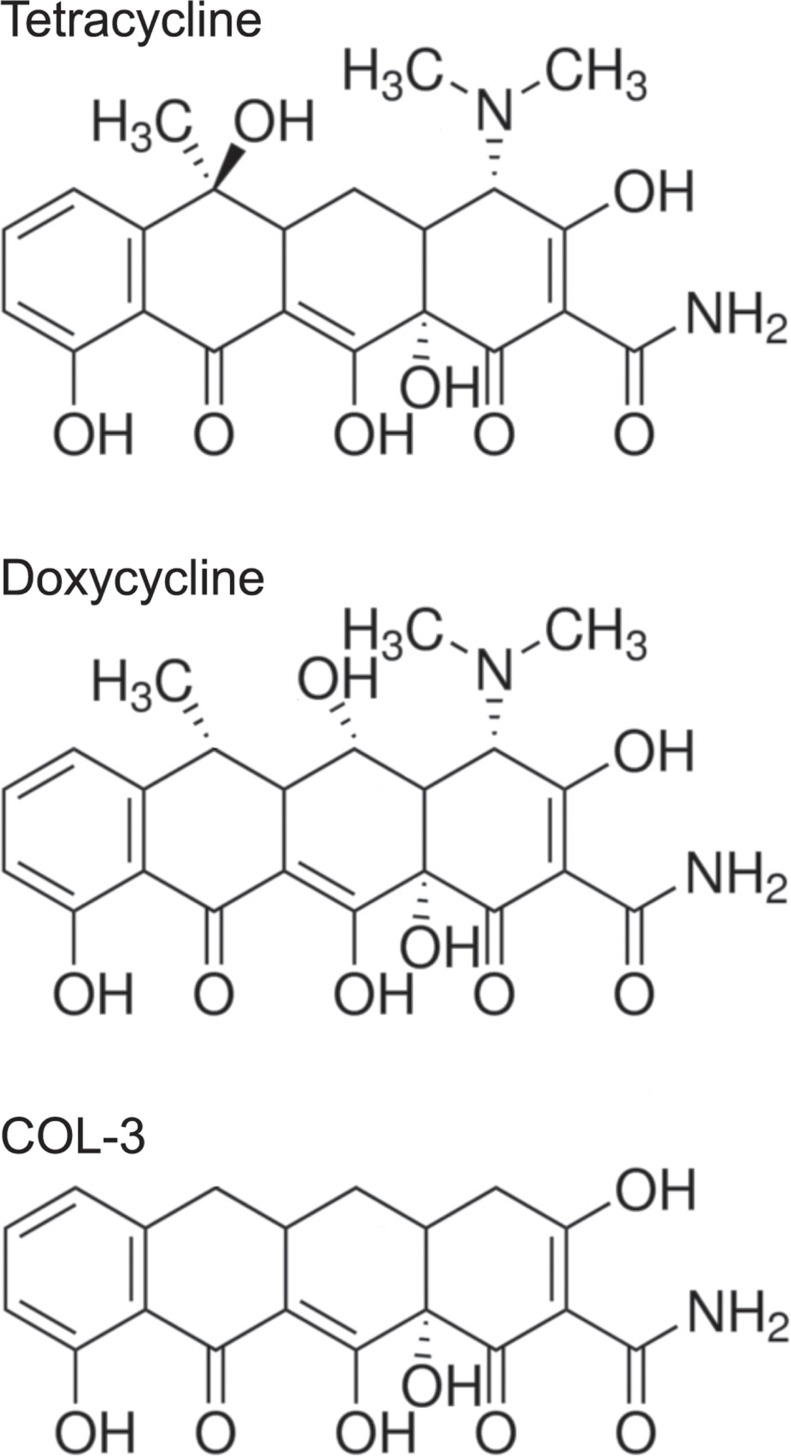
Chemical structures of tetracycline, and its analogs doxycycline and COL-3

Tetracyclines are antibiotics that inhibit bacterial protein synthesis by binding to the small ribosomal subunit and blocking the attachment of aminoacyl-tRNAs to the A site on the ribosome. Tetracyclines have an octahydronaphtacene structure consisting of four fused rings (Figure 1). The two long edges of this skeleton show a notable difference in the distribution of polar groups, producing a preference for hydrophobic interactions on one face and for hydrogen bonding on the other. This spatial arrangement is believed to be essential for the interaction with the small ribosomal subunit [1]. Doxycycline (DC; Figure 1) is one of the most commonly prescribed tetracycline derivatives. It is an inexpensive and safe drug, in use for more than 50 years to treat or prevent bacterial infections. It has optimal pharmacokinetic properties in humans and produces no bacterial resistance at a contra-indicative rate [2].

The potential use of tetracyclines in cancer therapy was first proposed in the 1980’s [3]. This proposal was based on the notion that mitochondrial ribosomes are evolutionarily related to bacterial ribosomes, whereas eukaryotic cytosolic ribosomes are evolutionary related to archeal ribosomes. The suggestion was supported by experiments with various cancer cell cultures and animal models, which demonstrated that tetracyclines exhibit anticancer properties. The effects were attributed to an inhibition of mitochondrial protein synthesis, which lead to a decrease of the mitochondrial energy generating capacity in proliferating cells and result in a proliferation arrest in the G_1_ phase of the cell cycle [4]. In later years, several other mitochondrial changes were reported to be induced by tetracyclines in tumor cells, including cytochrome-*c*-driven apoptosis, and a decrease in mitochondrial DNA-encoded proteins, respiration and inner membrane potential [5–9]. The postulated underlying bioenergetic mechanisms were reviewed recently [10].

Others have suggested that tetracyclines exert anticancer effects through their ability to chelate divalent cations, a property that is thought to inhibit matrix metalloproteinases (MMPs) that use zinc ions as a co-factor [11]. This led to the development of several new, chemically modified tetracyclines that lack the dimethylamino group considered crucial for the interaction with bacterial ribosomes, thus avoiding disruption of the gastrointestinal microbiota [12–15]. The most frequently studied tetracycline analogue void of antibacterial action is COL-3 (Figure 1). *In vitro* studies and work in animal models suggested that that COL-3 prevented, or at least restricted, metastasis and angiogenesis [16, 17]. COL-3 has also been tested in Phase I and II clinical trials with patients suffering from Kaposi sarcoma [18, 19], recurrent high-grade gliomas [20], and from various other advanced malignancies and refractory metastatic cancers [21, 22]. COL-3 is FDA-approved for chronic inflammatory periodontal and skin diseases [23]. The trials and approvals were all based on the claimed inhibitory effect of COL-3 on the activity of MMPs in tumor or inflamed stroma. Unfortunately, actual measurements of MMP enzyme activity were not performed in these studies. MMP activity assays were carried out in patients with abdominal aortic aneurysm treated with DC but failed to show differences compared to the untreated patient group [24]. In addition, the effects of any of the new, chemically modified tetracyclines on mitochondrial protein synthesis have not been investigated. We have argued that the serum and tissue levels of tetracycline analogs obtained with standard medication will not be sufficient to affect MMPs. Instead, we think that the obtained results should be interpreted to be caused by inhibition of clonal cell proliferation [25, 26].

It has been reported that COL-3 not only induces apoptosis but also necrosis [5, 27], a property not described for DC at therapeutic concentrations. In addition, leukopenia and thrombocytopenia were observed as side effects in a Phase I trial with COL-3 [22]. These adverse reactions and the fact that data on the effects of COL-3 on the expression of mitochondrially synthesized proteins are still lacking, prompted us to compare COL-3 with DC systematically in cell culture systems. Our experiments show that DC specifically affects mitochondrially encoded translation products, whereas COL-3 acts as a nonspecific inhibitor, affecting mitochondrially encoded proteins as well as nuclear-encoded proteins. DC remains the optimal choice for a tetracycline-based chemotherapy due to its more selective mitochondria-based mechanism of action.

## RESULTS

### Experimental outlay

First, we compared the cytotoxicity of COL-3 and DC after 5 days of treatment of human cell cultures in viability dose-response curves. This was followed by a comparison of the proliferation of cell cultures treated with a single concentration of the drugs over a 5-day period in time course cell growth curves. Next, we investigated the effect of the drugs on *de novo* mitochondrial protein synthesis, followed by an assessment of the levels and enzymatic activity of several mitochondrial proteins over a 5-day treatment period. Finally, we investigated whether cells treated with the drugs showed evidence of apoptosis.

### Cytotoxicity of COL-3 and DC

**Figure 2:**
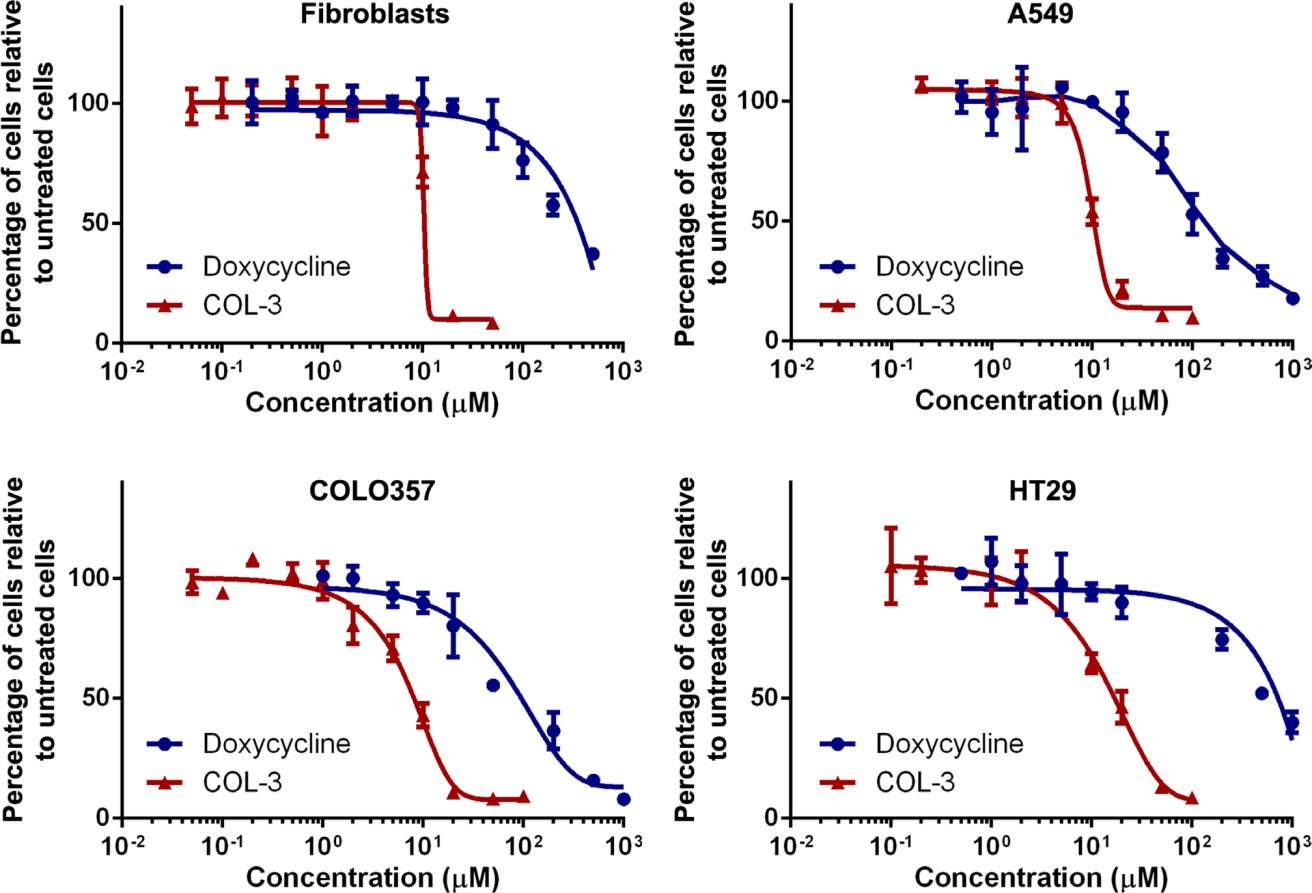
COL-3 is more cytotoxic than DC Viability dose-response curves of cultured fibroblasts, and the A549, COLO357 and HT29 cancer cell lines treated with doxycycline or COL-3 for 5 days. Experiments were performed in quadruplicate. Results are shown as mean percentage of surviving cells relative to vehicle-treated cells ± standard deviation.

The cytotoxicity of COL-3 and DC was compared in human cell cultures treated for 5 days with vehicle (DMSO) or serial dilutions of the drugs. We evaluated the A549 lung adenocarcinoma, the COLO357 pancreatic adenocarcinoma and the HT29 colon adenocarcinoma cell lines, and used primary fibroblast cultures as controls. The viability dose-response curves shown in Figure 2 clearly demonstrate that COL-3 is considerably more cytotoxic than DC. Toxicity for COL-3 is already apparent at concentrations that may be reached in clinical studies [20, 22]. The concentrations of the drugs resulting in a 50% growth inhibition over the 5-day treatment period are given in Table 1. The toxicity of COL-3 is 12 to 40-fold higher than DC. Intriguingly, HT29 cells were 6.0-fold more resistant to DC but only 1.8-fold more resistant to COL-3, compared to A549 cells. Fibroblasts were 2.6-fold more resistant to DC but about equally sensitive to COL-3 as A549 cells.

**Table 1:** Drug concentrations resulting in a 50% growth inhibition over a 5-d culture period

Cell culture	50% Growth inhibition	
	Doxycycline (µM)	COL-3 (µM)
Fibroblasts	310	10.5
A549	120	10.0
COLO357	100	8.3
HT29	715	18.1

### A549 cell proliferation in presence of COL-3 or DC

**Figure 3:**
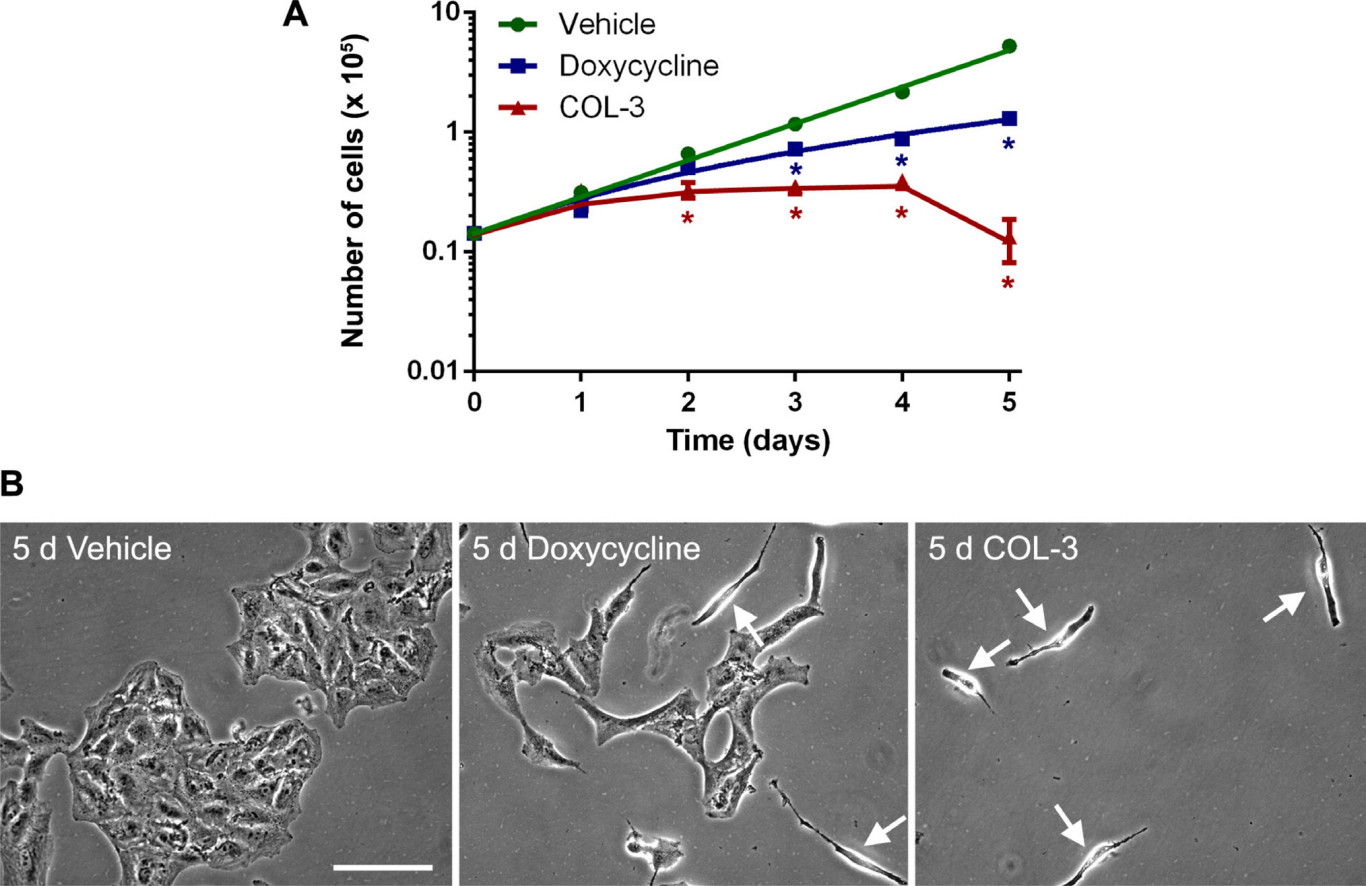
DC decreases the proliferation rate of A549 cells, whereas COL-3 kills A549 cells after 5 d of treatment (**A**) Time course proliferation curves of A549 cells treated with vehicle, 19.1 µM doxycycline, or 8.1 µM COL-3 over a 5-day time period. Experiments were performed in quadruplicate. Results are shown as mean number of cells ± standard deviation. Asterisks denote statistically significant differences compared to vehicle-treated cells (*p* < 0.05). (**B**) Micrographs of A549 cell cultures treated with vehicle, 19.1 µM doxycycline or 8.1 µM COL-3 for 5 d. Arrows indicate dying cells. Scale bar: 100 μm.

To look at the effect of the drugs on cell proliferation over a 5-day treatment period in more detail, we treated A549 cell cultures with 8.1 µM (3 μg/ml) of COL-3 or 19.5 µM (10 μg/ml) of DC or vehicle for 1 to 5 days and determined cell numbers each day. The concentrations of the drugs were chosen so that they correspond to the expected serum levels of COL-3 and DC *in vivo* in patients treated with the standard recommended dose [20, 22, 28, 29]. Results of the time course experiment are shown in Figure 3A. A549 cells treated with vehicle show logarithmic growth with a doubling time of 23.1 hour. The drugs have no effect on the cell proliferation rate after 1 day of treatment (approximately 1 doubling time) but further treatment with COL-3 halts proliferation completely and results in massive cell death during the final day of treatment. In contrast, further treatment with DC does not halt but slows the proliferation, especially after day 2 (approximately 2 doubling times). Thus, cells cultured in the presence of COL-3 show a lag phase of 1 doubling time before they respond to the drug, whereas cells cultured in the presence of DC show a lag phase of about 2 doubling times. Microscopic examination of the A549 cells after 5 days of treatment (Figure 3B) confirmed the slower growth of the cells treated with DC and revealed some cells in the process of dying. Five days of treatment with COL-3 resulted in a very sparse population of dying cells (Figure 3B).

### *De novo* mitochondrial protein synthesis in presence of COL-3 or DC

**Figure 4:**
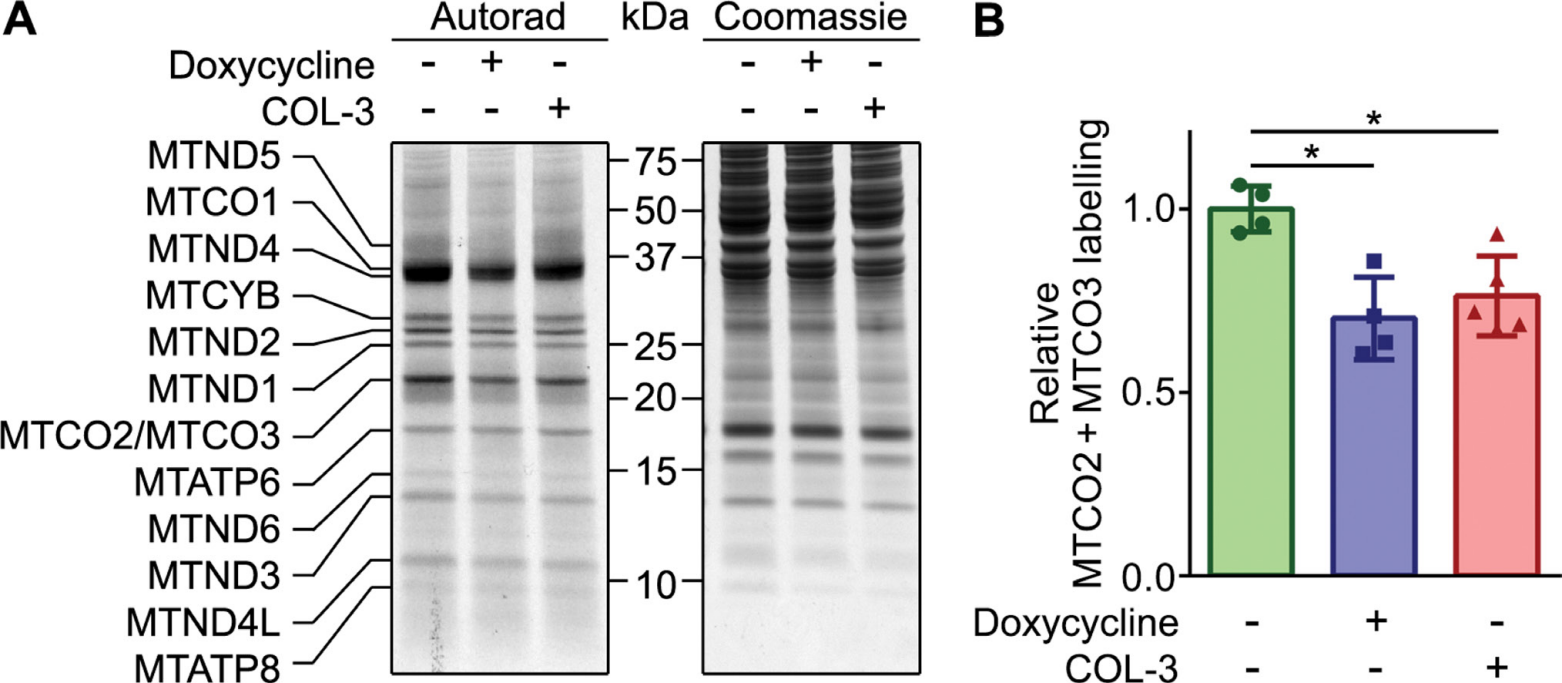
DC and COL-3 decrease mitochondrial translation (**A**) *De novo* mitochondrial protein synthesis of A549 cells treated with vehicle, 19.1 µM doxycycline, or 8.1 µM COL-3. Cells were pre-treated with the drugs for 1 hour, followed by a 1-hour pulse labeling with [^35^S]-methionine in the presence of the drugs and emetine to block cytosolic protein synthesis. Denatured, 30-μg protein samples were subjected to 12% acrylamide gel electrophoresis. The gel was stained with Coomassie to verify even loading, followed by autoradiography to reveal the labeled mitochondrial translation products, indicated on the left. The migration of protein standards is indicated in the center. (**B**) Mean MTCO2 + MTCO3 labeling signals of the drug-treated cells relative to the mean value of vehicle-treated cells of four independent experiments. Error bars indicate standard deviation. Asterisks denote statistically significant differences (*p* < 0.05).

As outlined in the introduction, we believe that the *in vivo* anticancer effect of DC is based on its inhibition of mitochondrial protein synthesis. We therefore compared the effect of COL-3 and DC on mitochondrial translation. For this purpose, A549 cell cultures were treated with 8.1 µM of COL-3 or 19.5 µM DC or vehicle for 1 h before and during a 1-h labeling with [^35^S]-methionine in the presence of emetine to inhibit cytosolic protein synthesis. Autoradiography of samples resolved by gel electrophoresis clearly revealed radioactive labeling of the 13 polypeptides encoded by mitochondrial DNA (Figure 4A). In addition, the autoradiographs showed that both drugs resulted in a general decrease of the *de novo* mitochondrial protein synthesis. Quantification of the radioactive signals of the co-migrating cytochrome-*c* oxidase subunits II and III (MTCO2 + MTCO3) from four independent experiments showed that the two drugs result in a similar decrease of mitochondrial protein synthesis of ∼27% compared to vehicle-treated cells (Figure 4B).

### The effect of COL-3 and DC on mitochondrial protein expression

**Figure 5:**
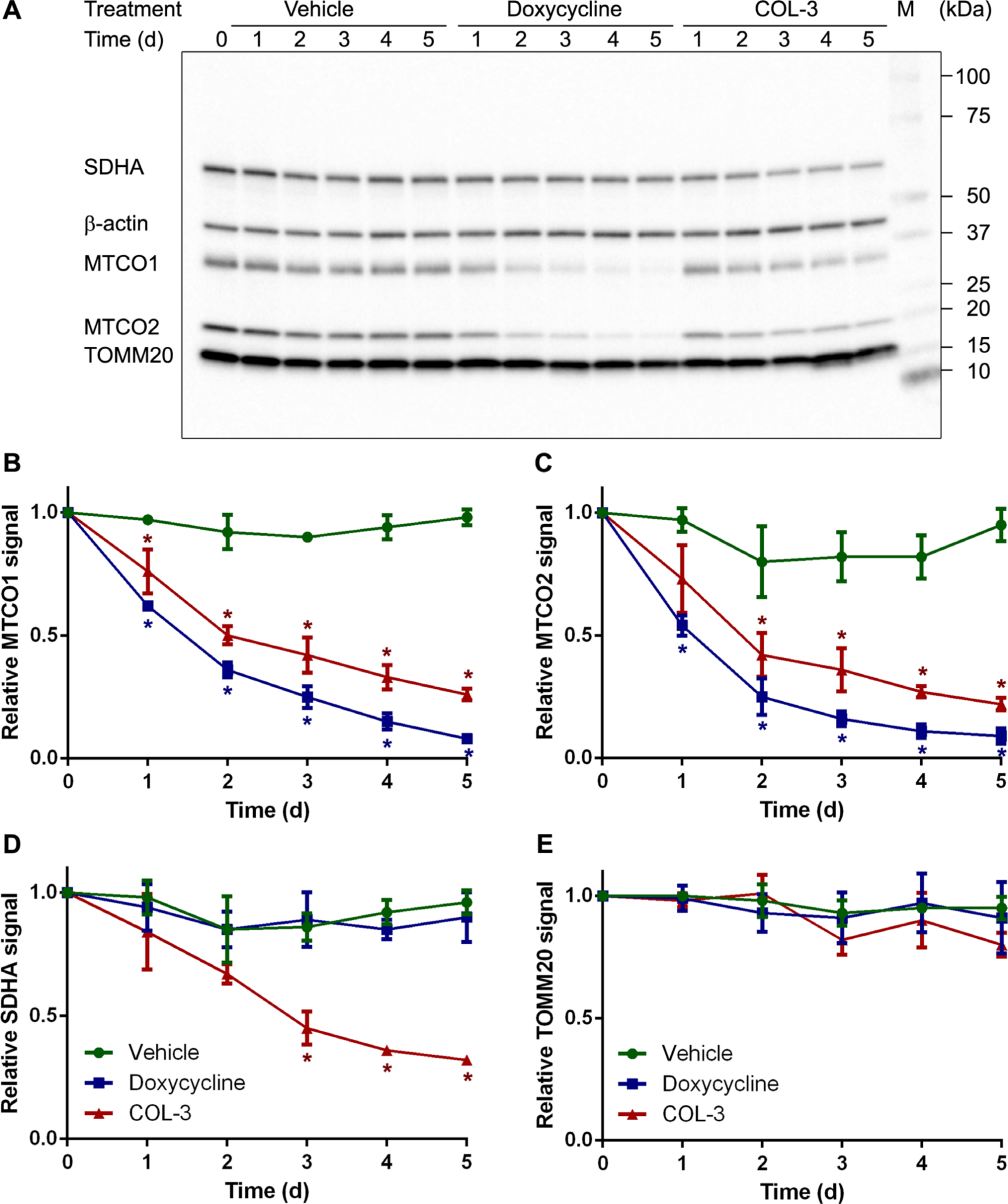
DC decreases the levels of mitochondrially synthesized proteins, whereas COL-3 decreases the levels of mitochondrially as well as cytosolically synthesized proteins (**A**) Western blot image of denatured, 10-μg protein samples from A549 cells treated with vehicle, 19.1 µM doxycycline, or 8.1 µM COL-3 over a 5-day time period and subjected to 4–20% gel electrophoresis. The blot was developed with antibodies raised against SDHA, MTCO1, MTCO2, TOMM20 and β-actin as loading control. The migration of a protein standard marker (M) is indicated on the right. (**B**−**E**) Mean MTCO1 (B), MTCO2 (C), SDHA (D) and TOMM20 (E) signals in the treated cells over a 5-day time period, relative to the signal of cells at t = 0 in four independent experiments. Error bars indicate standard deviation. Asterisks denote statistically significant differences from vehicle-treated cells (*p* < 0.05).

To investigate the specificity of the two drugs in more detail, we determined the levels of a number of mitochondrial proteins by western blot in A549 cells. The treatment protocol and chosen drug concentrations were identical to those of the time course proliferation study as shown in Figure 3. The western blots revealed that DC treatment resulted in a steady decrease of the two mitochondrially synthesized subunits of cytochrome-*c* oxidase MTCO1 and MTCO2 over the 5-day treatment period to ∼12% of control values. COL-3 treatment also resulted in a steady decrease, but the decline was not as strong as for DC (Figure 5A–5C). As anticipated, DC treatment had no effect on the levels of subunit A of succinate dehydrogenase (SDHA), which is synthesized in the cytosol; however, unexpectedly, 5-day treatment with COL-3 led to a 68% reduction of SDHA levels (Figure 5A, 5D). We also determined the levels of the mitochondrial outer membrane protein TOMM20 in surviving cells. This protein is synthesized in the cytosol and is a good indicator of the amount of mitochondria. Both DC and COL-3 treatment had no statistically significant effect on TOMM20 levels, suggesting that the total amount of mitochondria is not affected by the drugs (Figure 5A, 5E).

### The effect of COL-3 and DC on mitochondrial enzyme activity

**Figure 6:**
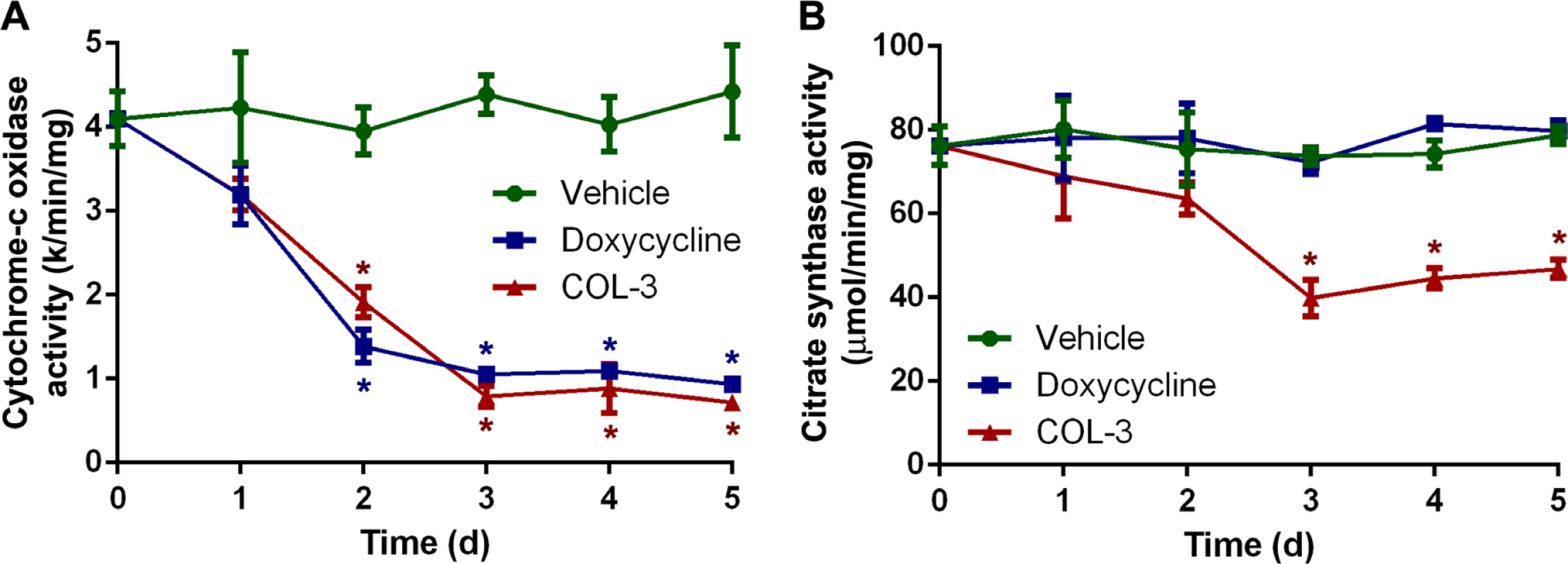
DC decreases the activity of cytochrome-*c* oxidase, whereas COL-3 decreases the activity of cytochrome-*c* oxidase as well as citrate synthase Cytochrome-*c* oxidase (**A**) and citrate synthase (**B**) activity in A549 cells treated with vehicle, 19.1 µM doxycycline, or 8.1 µM COL-3 over a 5-day time period. Assays were performed in quadruplicate. Results are shown as mean value ± standard deviation. Asterisks denote statistically significant differences from vehicle-treated cells (*p* < 0.05).

To investigate the functional implications of the decreased mitochondrial protein synthesis, we compared the effect of the two drugs on the activity of two mitochondrial enzymes: the respiratory chain enzyme cytochrome-*c* oxidase, which contains three subunits synthesized in mitochondria [30], and the Krebs cycle enzyme citrate synthase, which is constituted entirely of subunits synthesized in the cytosol. Again, the treatment protocol and chosen drug concentrations were identical to those in the time course proliferation study. The enzyme activity measurements revealed that both COL-3 and DC have a similar, strong effect on the cytochrome-*c* oxidase activity (Figure 6A). After ≥3 days of treatment, the activity is only ∼20% of that of the vehicle-treated cells. As expected, DC did not have any effect on citrate synthase activity but, remarkably, COL-3 treatment resulted in a >40% decrease in activity after ≥3 days of treatment (Figure 6B).

### Do COL-3 and DC cause apoptosis?

**Figure 7:**
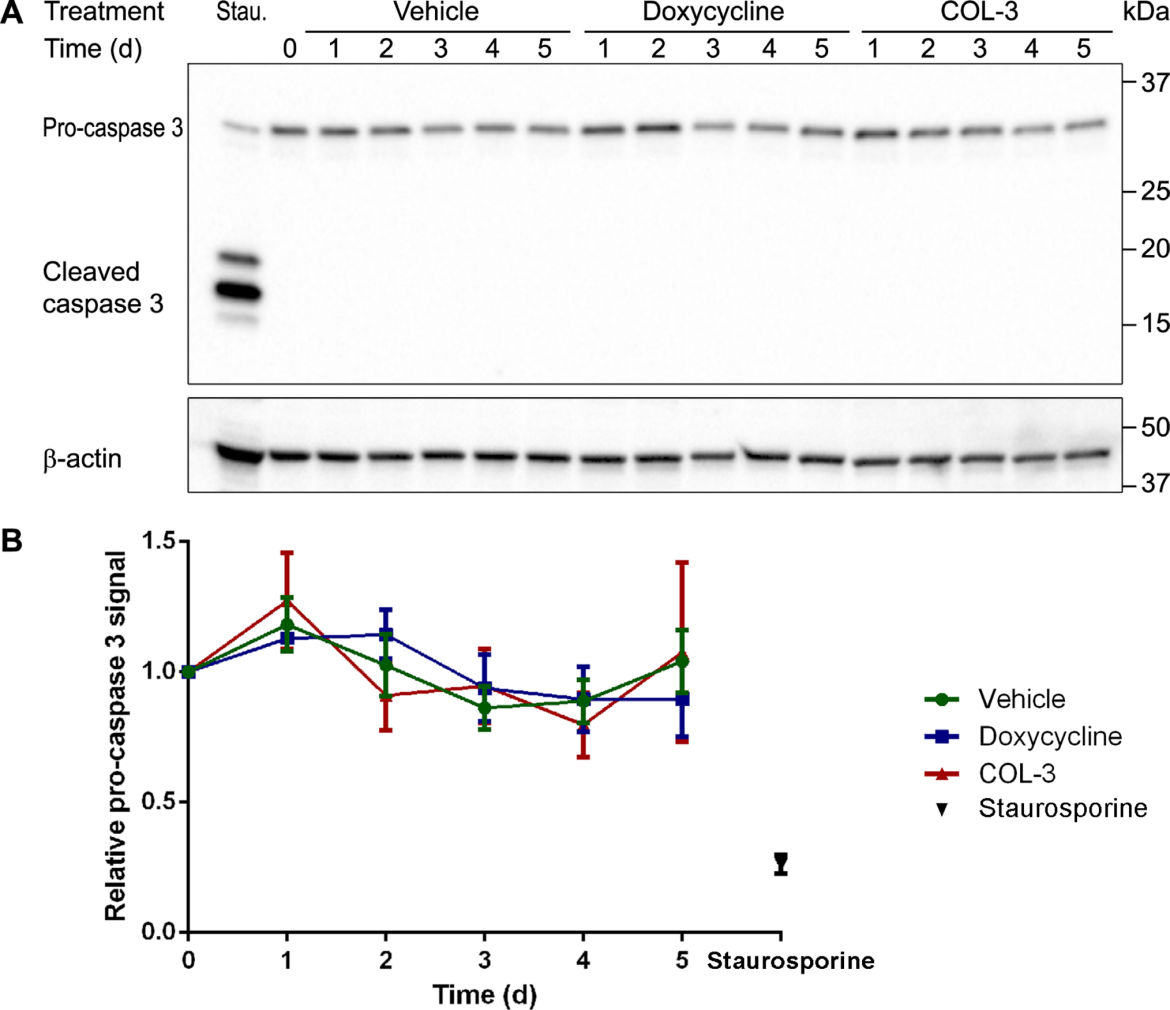
Absence of active cleaved caspase 3 indicates that DC and COL-3 do not induce apoptosis (**A**) Western blot image of denatured, 10-μg protein samples from A549 cells treated with vehicle, 19.1 µM doxycycline, or 8.1 µM COL-3 over a 5-day time period and subjected to 12% gel electrophoresis. A549 cells treated with 1 µM staurosporine (Stau.) for 3 h served as an apoptosis positive control sample. The blot was sequentially developed with antibodies raised against caspase 3 and β-actin as loading control. The migration of a protein standard marker is indicated on the right. (**B**) Mean procaspase 3 signals in the treated cells, relative to the signal of cells at t = 0 in three independent experiments. Error bars indicate standard deviation.

The time course experiments (Figure 3A) showed that COL-3 stops cell proliferation after 1 day of treatment and results in massive cell death at day 5, whereas DC slows down cell proliferation after 2 days of treatment but does not result in massive cell death. To investigate if the drugs cause apoptosis, we determined the levels of procaspase 3 and active (cleaved) caspase 3 over a 5-day treatment period on western blots. As a positive control, we used a sample of cultured A549 cells treated with the alkaloid staurosporine, which is known to induce apoptosis. Unlike in the staurosporine-treated cells, we observed no decrease in procaspase 3 levels in the COL-3 and DC-treated cells and, importantly, we did not detect cleaved caspase 3 over the 5-day treatment period (Figure 7). Because (cleaved) caspase 3 is the master coordinator of the destruction of cellular structures during apoptosis, the results indicate that neither COL-3 nor DC induce apoptosis. The massive cell death seen in COL-3-treated cells is, therefore, likely to be the result of necrosis. This is further supported by the absence of apoptotic bodies at day 5 (Figure 3B).

## DISCUSSION

The results of this study clearly confirmed that the main mode of action of DC is its inhibition of the expression of mitochondrially encoded translation products. These proteins are indispensable for oxidative phosphorylation and, thus, the mitochondrial energy generating capacity. In proliferating cells, this process can be inhibited without interfering with the expression of the nuclear-coded genetic information. In contrast, COL-3 appears to affect almost every aspect that we have investigated, including the mitochondrial protein synthesis, the levels of mitochondrially encoded (MTCO1 and MTCO2) as well as nuclear encoded (SDHA) proteins, and the activity of cytochrome-*c* oxidase, which contains mitochondrially synthesized subunits, as well as citrate synthase, which is composed solely of subunits synthesized in the cytosol. Structural differences between DC and COL-3 may alter other cellular mechanisms of toxicity, e.g. the assembly or function of the spliceosome [31], which is structurally similar to the ribosome. Tetracyclines may also alter gene splicing by directly interacting with pre-mRNA [31]. Subtle-differences in structure-activity relationships among tetracycline analogs may explain observed differences in the cell-specific cytotoxicity of COL-3 and the mechanism by which it alters the expression of nuclear encoded proteins like SDHA and citrate synthase.

In the viability dose-response experiments, 5-d treatment of A549 cells with 8.1-µM COL-3 resulted in a ∼26% decrease of cells compared to untreated cells (Figure 2), whereas this treatment resulted in a complete collapse of the culture in the proliferation time course experiments (Figure 3A). The cytotoxicity of 19.1 µM DC also appeared less in the dose-response than in the time course experiments with A549 cells. In the dose-response experiments, cells were cultured in 96-well plates to allow the measurement of the effect of a large number of drug concentrations, while cells were cultured in 10-cm plates in the time course experiments to permit the parallel determination of cell number (Figure 3A), protein level (Figures 5, 7) and enzyme activity (Figure 6). Apparently, these different experimental set-ups affect the cytotoxicity of the drugs.

A 5-day treatment of A549 cells with DC or COL-3 caused a striking decrease of the mitochondrially encoded cytochrome-*c* oxidase subunits MTCO1 and MTCO2, and cytochrome-*c* oxidase activity to 11–28% of control values, whereas a 2-h treatment with the drugs resulted in a relatively modest fall in *de novo* mitochondrial protein synthesis to ∼73% of control values (Figures 4–6). It may seem that this reduction in mitochondrial protein synthesis is too small to explain the dramatic decreases in MTCO1 and MTCO2 levels, and cytochrome-*c* oxidase activity but we expect that longer treatment with the drugs will lead to a larger drop in mitochondrial protein synthesis.

The doubling time of the three cancer cell lines that we investigated is very similar under untreated, basal growth conditions (∼24 h). We found that HT29 cells are more resistant to the drugs than A549 cells, especially to DC (Figure 2; Table 1). We assume that HT29 cells are not necessarily less sensitive to the anti-mitochondrial action of DC but that the antibiotic is imported less efficiently into the cells, or is eliminated more efficiently. The increased resistance of fibroblast to DC compared to A549 cells is expected in view of their longer doubling time of 43 h, resulting in a slower decrease of mitochondrially synthesized proteins through cell division. In contrast, fibroblasts and A549 cells are similarly sensitive to COL-3, suggesting that the toxicity of COL-3 involves an active mechanism in addition to the passive dilution of mitochondrially synthesized proteins through cell division.

### The lag phase

As shown in the viability dose-response curves (Figure 2), cell proliferation is less inhibited by DC than by COL-3 at moderate molarity. Proliferation time course experiments (Figure 3A) revealed that the lag phase, before an effect of 19.5 µM DC on the proliferation rate of A549 cells becomes apparent, is 2 days. By then, the levels of both the mitochondrially synthesized cytochrome-*c* oxidase subunits and cytochrome*-c* oxidase activity have decreased to about one-third of normal levels (Figures 5A–5C and 6). Apparently, at this point, the mitochondrial energy generating capacity is too low to sustain normal cell growth and, consequently, the proliferation rate drops. Although longer treatment with DC results in a further decrease of the mitochondrially synthesized subunits and cytochrome-*c* oxidase activity, the proliferation does not come to a complete halt within the 5-day time frame. This suggests that under the *in vitro* culturing conditions, the cells are still able to generate sufficient energy to sustain some proliferation. A somewhat stronger inhibition of the proliferation rate has been obtained by Onoda *et al.* [5], who performed cytotoxicity studies over a 2-d time period. Several groups have observed that cells treated with DC accumulate in the G_1_ phase of the cell cycle, prior to the S phase when the bulk of the building blocks for cell division are synthesized [4, 7, 32–34]. This is compatible with the idea that insufficient mitochondrial energy supply reduces the proliferation rate by impeding the synthesis of macromolecules.

The lag phase of 8.1-µM COL-3 treatment of A549 cells is only 1 day, which corresponds to the observation by Song *et al.* [8]. After 1 day of COL-3 treatment, proliferation of A549 cells completely stops. Also for COL-3, a modest arrest in G_0_/G_1_ has been reported in a short-term experiment [35]. This is in line with our observation of decreased mitochondrially synthesized protein levels and cytochrome-*c* oxidase activity. The much shorter lag phase and complete growth arrest by COL-3 may be explained by the fact that COL-3 causes a much broader spectrum of effects than DC, resulting not only in defects of mitochondrially synthesized cell components but also in cytosolically synthesized cell components, such as succinate dehydrogenase and citrate synthase. The longer lag phase seen in DC-treated cells strongly supports our view that the proliferation arrest occurs secondary to the inhibition of mitochondrial protein synthesis. It is also the basis for our opinion that mitochondria are a prime oncotarget. The usefulness of DC is based on the fact that normal tissues do not suffer from serious side effects because they are either post-mitotic or their proliferation rate tends to be relatively slow, their mitochondrial content is plentiful and they have ample mitochondrial energy generating capacity. This view is supported by the observed diminished DC cytotoxicity of primary fibroblast cultures compared to the A549 and COLO357 cell lines (Table 1). Notable exceptions to the slow proliferation of normal cells are the rapidly dividing hemopoietic stem and progenitor cells. However, tetracyclines do not appear to affect the proliferation of these cells from the bone marrow. Long-term continuous treatment of rats with oxytetracycline by intravenous transfusion revealed that the animals restored their anemia after bleeding as quickly as controls transfused with vehicle. Effects of oxytetracycline on immune responses were only found for events related to T-cell proliferation, not B-cell proliferation [36]. Apparently, the resistance of T-cells is changed during their passage through the thymus. In their recent publication, Pulvino *et al.* [37] report a cytostatic effect on doxycycline on diffuse large B-cell lymphoma and other non-Hodgkin lymphomas. Perhaps these cells underwent genetic changes during their oncogenic transformation that reversed their resistance to tetracyclines.

### Is there a need for tetracycline analogs void of antibacterial activity?

The claimed necessity for tetracycline analogs as anticancer drugs that are void of antibacterial activity underlies the development of chemically modified tetracyclines [11]. However, sound scientific considerations or experimental and medical evidence for this opinion are lacking. DC does not appear to provoke a serious resistance problem for the human microbiome. In fact, DC is seldom mentioned in publications were resistance data of tetracyclines are compared. Bartlett *et al*. [38] reported that tetracycline increases the occurrence of resistant *Escherichia coli* by 1000-fold in the fecal flora, compared to DC. The pharmacokinetic properties of DC are in this respect advantageous; at oral application, the rapid rise of serum levels indicates that the antibiotic is largely absorbed by the small intestine and, thus, DC represents a low burden for the colorectal tract, the main reservoir of gut microbes. Serious side effects of DC are also uncommon. Phototoxicity is mentioned as a side effect of DC. Treatments are, therefore, sometimes combined with a sunblock, although simply refraining from direct sunlight will suffice. Hyperpigmentation [39, 40] and photo-onycholysis [41] are reported sporadically but, altogether, there is little reason to exclude DC as a potential drug in the fight against cancer.

Our experiments show that COL-3 has kept the inhibitory effect on mitochondrial protein synthesis. For this reason, the loss of antibacterial properties of COL-3 cannot be attributed to the principle mode of action of tetracyclines at the level of protein synthesis. Most likely, the loss of antibacterial effects of COL-3 is based on its impermeability of the bacterial cell walls. The available evidence leads to the conclusion that DC is far more preferable than COL-3 to treat cancer, either on its own or in combination therapy. Clinical trials with COL-3 should be discouraged or, at least, be performed double-blind DC-controlled, rather than placebo-controlled.

## MATERIALS AND METHODS

### Cell culturing

A primary human dermal fibroblast culture was established from a skin explant of a 69-year-old male control subject after informed written consent, according to standard procedures. Ethical approval for this work was obtained from the Royal Free Hospital and Medical School Research Ethics Committee (REC 07/H0720/161). Fibroblasts, A549 human alveolar adenocarcinoma [42], COLO357 human pancreatic adenocarcinoma [43] and HT29 human colon epithelium adenocarcinoma [44] cell lines were all cultured in Dulbecco’s modified Eagle’s medium (DMEM) containing GlutaMAX™ and 4.5 g of glucose/l (Gibco Lifetechnologies, Cat. No.: 61965–029), and supplemented with 10% fetal bovine serum, 1 mM sodium pyruvate, 50 μg/ml of uridine, 50 units/ml of penicillin and 50 μg/ml of streptomycin at 37° C in a humidified atmosphere of 5% CO_2_ in 95% air, unless stated otherwise. The cancer cell lines were obtained from the European Collection of Authenticated Cultures and used for the experiments within two weeks after receipt.

### Determination of cytotoxicity after 5 days of treatment

To examine the cytotoxicity of DC and COL-3, cells were seeded in the wells of black Corning 96-well CellBIND^®^ assay plates with clear flat bottoms (Cat. No.: 3340) at an empirically derived density that would allow logarithmic cell growth over a 6-day time period. The next day, medium was changed and cells were treated with serial dilutions of DC, COL-3 or vehicle (dimethyl sulfoxide, DMSO), using stock solutions of 50 mM doxycycline hyclate (Sigma, Cat. No.: D-9891) in water and 20 mM COL-3 (trade name: Metastat; synonyms: CMT-3, 4-dedimethylaminosancycline, 6-demethyl-6-deoxy-4-dedimethylaminotetracycline; incyclinide; Echelon, Cat. No.: B-0802) in DMSO. Stocks were protected from light and stored at −20° C. Treatment was repeated each day over a 5-day time period. After the 5-day treatment, medium was removed and plates were frozen at −20° C. The number of cells per well was determined with the CyQUANT^®^ Cell Proliferation Assay Kit (ThermoFisher Scientific, Cat. No.: C7026), which measures the DNA content in a fluorescent assay on a plate reader. Cell numbers were calculated with the help of parallel measurements of serial dilutions of cell suspensions with a known cell concentration determined by trypan blue exclusion cell counting on C-Chip hemocytometer slides (NanoEnTek, Cat. No: DHC-N01). Experiments were carried out in quadruplicate and expressed as percentage of the number of cells in vehicle-treated wells.

### Determination of cell proliferation over 5 days of treatment

To assess the effect of DC and COL-3 on A549 cell proliferation over a 5-day treatment period, 1.0 × 10^4^ A549 cells were seeded on 10-cm tissue culture dishes (Sarstedt, Cat. No.: 83.3902). This seeding density allows logarithmic growth over a 6-day time period. The next day, medium was removed and cells were treated with 19.5 µM DC (10 μg DC per ml of medium), 8.1 µM COL-3 (3 μg COL-3 per ml of medium) or vehicle (0.4 µl DMSO per ml of medium) in fresh medium. Treatment was repeated each day over a 5-day time period, but every day dishes were chosen randomly to determine the number of cells per plate. Cells on these plates were dislodged by trypsinization, collected by centrifugation, resuspended and counted on C-Chip hemocytometer slides. Experiments were performed in quadruplicate with independent samples and expressed as absolute cell number. The population doubling time (DT) was calculated using the formula DT = (t-t_0_)log2/(logN-logN_0_), where t and t_0_ are the times at which the cells were counted, and N and N_0_ are the cell numbers at these times.

After 5 d of treatment, cell cultures were examined under an Olympus CK2 phase contrast microscope at 200× magnification. Images were captured with a Zeiss Axiocam MRm camera and were processed with Adobe^®^ Photoshop^®^ software.

### Labeling of mitochondrial translation products

To examine the effect of the drugs on *de novo* mitochondrial protein synthesis, A549 cells were cultured in wells of a Sarstedt 6-well cell culture plate (Cat. No.: 83.3920) until 80% confluency was reached. Then, cells were washed three times for 10 min with methionine and cysteine deficient DMEM (Gibco Lifetechnologies, Cat. No.: 21013–024), supplemented with 19.5 µM DC, 8.1 µM COL-3 or vehicle. Next, cells were incubated in methionine and cysteine deficient DMEM, supplemented with 0.5 mM L-cysteine, 2 mM L-glutamine, 1 mM sodium pyruvate, 5% dialyzed fetal bovine serum, and 19.5 µM DC, 8.1 µM COL-3 or vehicle for 10 min. Emetine dihydrochloride hydrate (Sigma, Cat. No.: E2375) was added to a final concentration of 100 μg per ml of medium and cells were incubated for a further 20 min. L-[^35^S]-Methionine (Perkin Elmer, Cat. No.: NEG009L010MC) was added to a final concentration of 5.3 MBq per ml of medium and cells were incubated for an additional 1 h. After 1 h, medium was removed, and cells were rinsed with phosphate-buffered saline (PBS) and dislodged with trypsin. Trypsinization was stopped with standard DMEM culture medium, and cells were collected by centrifugation at 1000 × *g* for 5 min. The cell pellet was washed once with PBS and then lysed with 0.1% *n*-dodecyl-β-D-maltoside (Anatrace; Cat. No.: D310) in PBS, 1 mM phenylmethylsulfonyl, 1 µg/ml of leupeptin 1 µg/ml of pepstatin A and 10 µl/ml of Benzonase^®^ nuclease solution (Sigma, Cat. No.: E1014) on ice for 15 min. Sodium dodecyl sulfate (SDS) was added to a final concentration of 1%, followed by a further 15-min incubation on ice. Then, samples were brought to room temperature to allow all SDS to dissolve and tubes were centrifuged at 12,000 × *g* for 5 min. The protein concentration of the supernatants was determined with the Pierce^®^ BCA Protein Assay Kit (ThermoFisher Scientific, Cat. No.: 23225), according to the manufacturer’s microplate procedure. Samples of 30 μg protein in 1× Laemmli Sample Buffer (BioRad, Cat. No.: 161–0747) and 1× NuPAGE Sample Reducing Agent (Lifetechnologies, Cat. No.: NP00009) were resolved by electrophoresis through NuPAGE 12% Bis-Tris Protein Gels (Lifetechnologies, Cat. No.: NP0341) with NuPAGE MES SDS Running Buffer (Lifetechnologies, Cat. No.: NP0002) alongside Precision Plus Protein™ Unstained Standards (BioRad, Cat. No.: 161–0373). After electrophoresis, gels were stained for 30 min with 0.25% Coomassie Brilliant Blue R-250 in 50% methanol and 10% acetic acid. The gels were destained for 5 h in 50% methanol and 10% acetic acid, followed by equilibration in 30% methanol, 5% glycerol overnight. Gels were dried under vacuum for 3 h at 70° C, followed by exposure to CL-XPosure film (Thermoscientific, Cat. No.: 34090). Images of the stained gels and autorads were captured with the BioRad Chemidoc™ MP Imaging System. Signals were quantified with Image Lab 5.1 software (BioRad). Radioactive signals on the autorad were normalized with the aid of the total Coomassie staining signal of the gel lanes and expressed relative to the radioactive signal from cells treated with vehicle. The experiments were performed in quadruplicate with independent samples.

### Determination of mitochondrial protein levels over 5 days of treatment

To evaluate the effect of DC and COL-3 on the expression of specific A549 mitochondrial proteins over a 5-day treatment period, 1.0 × 10^4^ A549 cells were seeded on 10-cm tissue culture dishes. The next 5 days, cells were treated daily with 19.5 µM DC, 8.1 µM COL-3 or vehicle, as described above for the time course proliferation assay. During the 5-day treatment, dishes were chosen randomly each day for harvesting of cells by trypsinization. Cells were collected by centrifugation in culture medium, washed twice with PBS and proteins were extracted with 1% Triton X-100 in PBS, 1 mM phenylmethylsulfonyl, 1 μg/ml of leupeptin and 1 μg/ml of pepstatin A on ice for 15 min. After centrifugation at 16,000 × *g*, 4° C, for 10 min, the protein concentration in the supernatants was determined with the Pierce^®^ BCA Protein Assay Kit. Supernatants were stored at -80° C and used within 1 month. To prepare western blots, 10-μg protein samples in 1× Laemmli Sample Buffer and 1× NuPAGE Sample Reducing Agent were resolved on Criterion™ TGX Stain-Free 4–20% precast gels (BioRad, Cat. No.: 5678094) alongside Precision Plus Protein™ Standards (BioRad, Cat. No.: 161–0374) and blotted onto Trans-Blot^®^ Turbo™ 0.2-μm PVDF membranes (BioRad, Cat. No.: 170–4157), using the BioRad Trans-Blot^®^ Turbo™ Transfer System. Protein binding sites on the blots were saturated with 10% skimmed milk powder in PBS for 1 hour, followed by a rinse with PBS, 0.3% Tween-20 and primary antibody incubation in PBS, 0.3% Tween-20, at 4° C, overnight. A cocktail of the following antibodies were used: anti-succinate dehydrogenase subunit A (SDHA; Abcam, Cat. No: ab14715), anti-cytochrome-*c* oxidase subunit I (MTCO1; Abcam, Cat. No.: ab140705), anti-cytochrome-*c* oxidase subunit II (MTCO2; Abcam, Cat. No.: ab110258), anti-translocase of outer mitochondrial membrane subunit 20 (TOMM20; Santa Cruz Biotechnology, Cat. No.: sc-11415) and anti-β-actin (Abcam, Cat. No.: ab6276). Excess of primary antibodies were removed with three 10-min washes in PBS, 0.3% Tween-20, followed by a 1-h incubation with the horse radish peroxidase-conjugated secondary antibodies goat anti-mouse IgG and goat anti-rabbit IgG (Dako, Cat. No.: P0447 and P0448) in PBS, 0.3% tween-20, and another three 10-min washes. Blots were developed with Clarity™ Western ECL Substrate (BioRad, Cat. No.: 170–5060). Capturing of the chemiluminescent signals was performed with the BioRad Chemidoc™ MP Imaging System. Signals were quantified with Image Lab 5.1 software. Mitochondrial protein signals were normalized with the aid of the β-actin signal and expressed relative to the signal from cells at t = 0. The experiments were performed in quadruplicate with independent samples.

### Determination of enzyme activities over 5 days of treatment

To investigate the effect of the drugs on A549 cytochrome-*c* oxidase and citrate synthase activity over a 5-day treatment period, 1.0 × 10^4^ A549 cells were seeded on 10-cm tissue culture dishes. The next 5 days, cells were treated daily with 19.5 µM DC, 8.1 µM COL-3 or vehicle, and samples were collected exactly as described above for the western blot experiments. Proteins were extracted with 1.5% *n*-dodecyl-β-D-maltoside in PBS, 1 mM phenylmethylsulfonyl, 1 μg/ml of leupeptin and 1 μg/ml of pepstatin A on ice for 15 min [45]. After centrifugation at 16,000 × *g*, 4° C, for 10 min, supernatants were stored at −80° C. The stored supernatants were used in the assays within 10 days of storage. All assays were carried out in quadruplicate.

Cytochrome-*c* oxidase activity was determined spectrophotometrically at 30° C by monitoring the rate of ferrocytochrome-*c* oxidation of 50 µM horse heart ferrocytochrome-*c* in 10 mM KP_i_ (pH 7.0) at 550 nm. The activity was calculated as the first-order velocity constant k [46]. Ferrocytochrome-*c* was prepared by reduction of 1 g of horse heart (ferri)cytochrome-*c* (Sigma; Cat. No.: C7752) with 13 mg of L-ascorbic acid in 100 ml of 100 mM KP_i_ (pH 7.0), followed by extensive dialysis in 10 mM KP_i_ (pH 7.0) at 4° C, and was stored at −80° C. Citrate synthase activity was measured according to Srere [47] at 30° C by monitoring the rate of 5-thio-2-nitrobenzoic acid formation of 100 µM 5,5′-dithiobis(2-nitrobenzoic acid) (Sigma, Cat. No.: D8130) in 100 mM Tris·HCl (pH 8.0), 100 µM acetyl Co-enzyme A (Sigma, Cat. No.: A2056), 100 µM oxaloacetic acid (Sigma, Cat. No.: O4126) and 0.1% Triton X-100, using a plate reader at 412 nm. The protein concentration of the samples was determined with the Pierce^®^ BCA Protein Assay Kit.

### Determination of caspase 3 levels over 5 days of treatment

To assess procaspase 3 and caspase 3 expression over a 5-day treatment period, the same samples as for the determination of mitochondrial protein levels were used. To obtain an apoptosis positive control sample, cultured A549 cells were treated with 1 µM staurosporine for 3 h, using a stock solution of 1 mM staurosporine (Sigma, Cat. No.: S4400) in DMSO. After treatment, cells were harvested and extracted with Triton X-100 as described above. Western blots were prepared as for the analysis of mitochondrial proteins, except that samples were resolved on Criterion™ TGX Stain-Free 12% precast gels (BioRad, Cat. No.: 568044). Protein binding sites on the blots were saturated with 1× Casein Blocking Buffer (Sigma, Cat. No.: B6429), followed by incubation with an anti-caspase 3 antibody (Cell Signaling, Cat. No.: 9662) in the same buffer at 4° C, overnight. Further washings, secondary antibody incubation and chemiluminescent imaging were carried out as described above. The blots were re-probed with an anti-β-actin antibody (Sigma, Cat. No.: A2066). The procaspase 3 signal was normalized with the help of the β-actin signal and expressed relative to the signal from cells at t = 0. Experiments were performed in triplicate with independent samples.

### Statistical analyses

All graphs and statistical analyses were executed with GraphPad Prism^®^ version 6.01 software. As the sample size was too small to confirm normal distribution, we used non-parametric Kruskal-Wallis tests to examine statistical significance. Statistical significance levels were set to *p* < 0.05 with Bonferroni correction for multiple pairwise comparisons.

## Abbreviations

DC: doxycycline
DMEM: Dulbecco’s modified Eagle’s medium
DMSO: dimethyl sulfoxide
MMP: matrix metalloproteinase
MTCO1–3: cytochrome-*c* oxidase subunits I–III
PBS: phosphate-buffered saline
SDHA: succinate dehydrogenase subunit A
SDS: sodium dodecyl sulfate
TOMM20: translocase of outer mitochondrial membrane 20
